# Identification of high-risk subgroups in very elderly intensive care unit patients

**DOI:** 10.1186/cc5716

**Published:** 2007-03-08

**Authors:** Sophia E de Rooij, Ameen Abu-Hanna, Marcel Levi, Evert de Jonge

**Affiliations:** 1 Department of Geriatrics, Academic Medical Center, University of Amsterdam, Meibergdreef 9 1105 AZ, Amsterdam, The Netherlands; 2 Department of Medical Informatics, Academic Medical Center, University of Amsterdam, Meibergdreef 9 1105 AZ, Amsterdam, The Netherlands; 3 Department of Internal Medicine, Cardiology and Pulmonary Disease, Academic Medical Center, University of Amsterdam, Meibergdreef 9 1105 AZ, Amsterdam, The Netherlands; 4 Department of Intensive Care, Academic Medical Center, University of Amsterdam, Meibergdreef 9 1105 AZ, Amsterdam, The Netherlands

## Abstract

**Introduction:**

Current prognostic models for intensive care unit (ICU) patients have not been specifically developed or validated in the very elderly. The aim of this study was to develop a prognostic model for ICU patients 80 years old or older to predict in-hospital mortality by means of data obtained within 24 hours after ICU admission. Aside from having good overall performance, the model was designed to reliably and specifically identify subgroups at very high risk of dying.

**Methods:**

A total of 6,867 consecutive patients 80 years old or older from 21 Dutch ICUs were studied. Data necessary to calculate the Glasgow Coma Scale, Acute Physiology and Chronic Health Evaluation II, Simplified Acute Physiology Score II (SAPS II), Mortality Probability Models II scores, and ICU and hospital survival were recorded. Data were randomly divided into a developmental (*n *= 4,587) and a validation (*n *= 2,289) set. By means of recursive partitioning analysis, a classification tree predicting in-hospital mortality was developed. This model was compared with the original SAPS II model and with the SAPS II model after recalibration for very elderly ICU patients in the Netherlands.

**Results:**

Overall performance measured by the area under the receiver operating characteristic curve and by the Brier score was similar for the classification tree, the original SAPS II model, and the recalibrated SAPS II model. The tree identified most patients with very high risk of mortality (9.2% of patients versus 8.9% for the original SAPS II and 5.9% for the recalibrated SAPS II had a risk of more than 80%). With a cut-point at a risk of 80%, the positive predictive values were 0.88 for the tree, 0.83 for the original SAPS II, and 0.87 for the recalibrated SAPS II.

**Conclusion:**

Prognostic models with good overall performance may also reliably identify subgroups of very elderly ICU patients who have a very high risk of dying before hospital discharge. The classification tree has the advantage of identifying the separate factors contributing to bad outcome and of using few variables. Up to 9.5% of patients were found to have a risk to die of more than 85%.

## Introduction

The number of very elderly patients in the population has grown rapidly and in the coming decades will continue to increase even further [1]. At present, this aging is associated both with an increased prevalence of comorbidities and functional disabilities and with an increasing need for intensive care facilities. There is much uncertainty regarding which very elderly patients will benefit from intensive care unit (ICU) treatment and which subgroups may be identified as having very low or high risks of mortality.

Prognostic models such as the Acute Physiology and Chronic Health Evaluation (APACHE) II or III [2,3], the Simplified Acute Physiology Score II (SAPS II) [4], and the Mortality Probability Models II (MPM II) [5] were developed to quantify the severity of illness and the likelihood of hospital survival for a general ICU population. These models should reliably predict the probability of mortality in all patients. However, little is known about the performance of these models in specific populations such as the very elderly. In addition, finding subgroups of very elderly patients who have a very high risk of dying may be important for several reasons. It identifies patients for whom better treatments are needed. At the same time, it may provide information to help patients and their caregivers to decide on intensive treatments that may be very burdensome. To decide on their willingness to receive intensive care treatment, very elderly patients want to know whether they have a fair chance of surviving [6,7]. Also, identification of high-risk groups of patients may be useful for risk stratification in scientific trials or for comparing outcomes of different ICUs.

The aim of our study was to develop a prognostic model for very elderly ICU patients 80 years old or older which could reliably identify patients at very high risk of death before hospital discharge. To develop such a model, we used two statistical methods, namely a recalibrated SAPS II model based on logistic regression and the technique of recursive partitioning analysis (RPA). RPA is a non-parametric technique that iteratively subdivides a population into subgroups by creating mutually exclusive subsets according to a set of predictor variables. The process results in a classification tree.

## Materials and methods

### Participants

We retrospectively studied 6,867 consecutive patients 80 years old or older admitted from January 1997 to December 2003 to the ICUs of 21 university, teaching, and non-teaching hospitals in the Netherlands. The data were obtained from the database of the Dutch National Intensive Care Evaluation (NICE) [8]. For the data analysis with recursive partitioning in this study, we randomly divided the data into a developmental (*n *= 4,578) and a validation (*n *= 2,289) set. The study was approved by the medical ethics committee of our hospital, a tertiary university hospital.

### Data collection

Data were collected as part of the NICE registry. For all patients, demographics, all the data necessary to calculate the Glasgow Coma Scale (GCS), APACHE II [2], SAPS II [4], and MPM II [5] scores, and ICU and hospital survival were recorded. So that reliable data can be collected, NICE incorporates a framework of measures to improve data quality. Details concerning the quality of the data used in this study have been published elsewhere [9].

### Missing data

There were 7,019 consecutive admissions in total. Records with missing values for admission type (*n *= 142, of which 47 resulted in death) and SAPS II scores (*n *= 10) were excluded from the analysis, resulting in 6,867 admissions. GCS had 977 missing values; these were considered to be normal (value = 15) and were therefore imputed in the training and the validation sets. The percentage of missing values of other relevant variables varied from 0% to 10%: urine production within 24 hours (*n *= 310), lowest bicarbonate (*n *= 536), urea (*n *= 693), mechanical ventilation within 24 hours after admission (*n *= 0), lowest systolic blood pressure (*n *= 214), and lowest pH (*n *= 670). The tree-fitting algorithm automatically handled missing values as described below.

### Statistical analysis

For continuous variables, we used the *t *distribution for calculating the 95% confidence intervals (CIs) and the Welch modification of the two-sample *t *test for calculating the *p *values for differences between means. This modification allows one not to assume equal variance in the survival and non-survival groups. We used Wilson's method for calculating the 95% CI for proportions and binomial probabilities such as mortality rate in the various patient subgroups and the positive predictive values (PPVs). The two-sided proportion test with Yates' continuity correction was used for testing differences between proportions (except for differences between PPVs, for which bootstrapping [with 1,000 bootstrap samples] was used because the patient groups partially overlap). Bootstrapping with 1,000 bootstrap samples was also used to calculate the CI of differences between Brier scores. The Hosmer-Lemeshow test with 10 degrees of freedom was used for testing model calibration.

In this study, data were analyzed by means of RPA, among other methods [10]. RPA is an alternative to more standard model-based regression techniques for multivariable analyses. In contrast to such numeric-based techniques, RPA results in a symbolic representation called a classification tree, which can be easily interpreted as a collection of 'if-then rules,' each with a condition part and a conclusion part. An example of a rule is 'IF the GCS score is greater than 6 AND the patient is admitted to the ICU after planned surgery AND the urine production during the first 24 hours is more than 1.25 liters, THEN the risk to die before hospital discharge is 11.8%.' The classification tree is obtained by finding the split – a variable and its value or cut-point value (for example, GCS score of more than 6) – that 'best' partitions the whole group of patients into two subgroups. These subgroups, one fulfilling and one not fulfilling the condition in the split, appear graphically under a left and a right branch emanating from the group.

The term 'best' refers to a partition resulting in the lowest entropy, meaning essentially that a probability of an event (such as survival status) differs most between the two subgroups. Next, each subgroup in turn is itself further partitioned (hence the term 'recursive partitioning' in RPA). This process is repeated until a stopping criterion is met. Each path from the root to a leaf node in the tree corresponds to an if-then rule in which the conclusion part consists of the probability of the event in the leaf node.

When the tree algorithm finds the split that best partitions a group of observations, it also identifies 'surrogate splits' used to handle missing values. A surrogate split partitions the observations in a way very similar to the original split (in terms of the 'left' and the 'right' subgroups). Suppose that the original split is 'minimum bicarbonate of less than 22.6 μmol/l'; for an observation missing the minimum bicarbonate value, the surrogate split 'maximum bicarbonate of less than 25.3 μmol/l' can be used to decide on whether the observation should go to the left or to the right branch. The surrogate-split mechanism is, in effect, a flexible way to impute a missing value depending on where it is encountered in the tree.

The surrogate splitter contains information that typically is similar to what would be found in the primary splitter. In our study, the root of the tree corresponds to the whole developmental sample and is associated with the prevalence (the *a priori *probability) of hospital mortality in the developmental set. Each variable is then assessed to determine which one discriminates most (in terms of information gain) between those who are discharged from hospital alive and those who did not survive hospital treatment.

This process is repeated on the new nodes, creating a tree structure as a result. This process was first allowed to completely overgrow the tree to overfit the data. Then the optimal tree size was determined as the size that results in the minimum cross-validation error, as described below. Then the original overgrown tree was pruned back to the optimal size.

Cross-validation is performed for increasing tree sizes (in essence, this corresponds to the number of nodes in the tree). The cross-validation error is based on a 10-fold cross-validation in which the developmental set is randomly split into 10 mutually exclusive subsets. Nine sets are used to grow a new tree of the given size, and the 10th is used to assess the accuracy of that tree in predicting the outcomes in this 10th subset. This process is repeated for each of the remaining nine sets to assess the performance, resulting in ten error estimates. The cross-validation error associated with the given tree size is the average value of the classification error of the 10 trees of that size. The cross-validation error will usually first decrease with tree size, then reach a minimum that is associated with the optimal tree size, but then start increasing again due to overfitting.

The resulting pruned tree was then validated by measuring its predictive performance on the validation set, which was not used in any way during the development of the tree. We used systematic sampling, including every third successive admission in the validation set.

We used the Rpart package for recursive partitioning [11] and the generalized linear model function for fitting logistic regression models within the statistical environment S-PLUS (commercially available software, Insightful Corporation, Seattle, USA) [12].

The predictive ability of the tree was compared with the predicted mortality based on the original SAPS II score and with the predicted mortality based on the SAPS II model after first-level customization for a Dutch population of very elderly patients by means of the developmental database [13]. First-level customization means refitting the model to obtain new coefficients without changing the score itself. Second-level customization implies adapting each item of the score; this was not attempted here. A receiver operating characteristic (ROC) curve was generated for the logistic regression SAPS II models and the classification tree. The ROC curve is a graphical display of sensitivity plotted against 1 – specificity for all possible thresholds that can be used to predict hospital mortality. Estimates of the area under the ROC curve (ROC-AUC) and its standard error were obtained using the non-parametric approach of DeLong and colleagues [14]. The ROC-AUC measures the discriminative ability of a model. It is not, strictly speaking, a proper scoring rule; that is, its maximum value can also be obtained when the predictions are not equal to the true probabilities. This is because it is not sensitive to the distance between the predicted probability and the true probability of an event, which is a measure of calibration. Therefore, we also measured the Brier score (that is, the mean of the squared errors of the predictions), which is a proper scoring rule. We also performed a Hosmer-Lemeshow test with 10 degrees of freedom.

## Results

The overall mortality was *n *= 1,433 (31.3%) of the developmental set and *n *= 699 (30.5%) of the validation set (difference not significant). The studied cohort had a mean age of 83.4 years (developmental set 83.3 years, validation set 83.5 years, difference not significant). Characteristics of patients are shown in Tables 1 and 2.

**Table 1:** Characteristics of patients surviving or not surviving until hospital discharge (developmental set)

Variable	Survivors (*n *= 3,145)	Non-survivors (*n *= 1,433)	*p *value^a^
Age in years^b^	83.2 ± 3.3	83.6 ± 3.4	< 0.05
Male	45.7%	47.8%	0.554
Maximum temperature in degrees Celsius^b^	37.5 ± 0.82	37.4 ± 1.37	0.397
Heart rate in beats per minute^b^	69.6 ± 16.7	67.4 ± 29.1	0.0107
Sodium in millimoles per liter^b^	137 ± 4.5	137 ± 5.87	0.00329
Potassium in millimoles per liter^b^	3.7 ± 0.544	3.78 ± 0.739	< 0.001
Creatinine in micromoles per liter^b^	104 ± 71.8	143 ± 105	< 0.001
Bicarbonate in millimoles per liter^b^	21.4 ± 4.02	18.7 ± 5.53	< 0.001
Albumin in grams per liter^b^	22.3 ± 6.8	20 ± 7.71	< 0.001
pH^b^	7.38 ± 0.0837	7.32 ± 0.124	< 0.001
Urine output in liters per 24 hours^b^	2.65 ± 2.02	1.75 ± 1.78	< 0.001
Glasgow Coma Scale			
Score = 15	2,950	1,029	< 0.05
Score less than 15	195 (6.2%)	404 (28.2%)	
APACHE II score^b^	16.7 ± 5.46	22.5 ± 8.16	< 0.001
APACHE II predicted mortality^b^	0.197 ± 0.178	0.432 ± 0.262	< 0.001
SAPS II score^b^	36.2 ± 12.1	54.2 ± 20.4	< 0.001
SAPS II predicted mortality^b^	0.219 ± 0.193	0.513 ± 0.305	< 0.001
Cardiopulmonary resuscitation before admission	2.7%	15.2%	< 0.001
Length of stay at intensive care unit in days^c^	1.0 (0.8–2.9)	1.9 (0.7–5.7)	< 0.001
Length of stay at hospital in days^c^	14 (9–25)	10 (3.2–24)	0.04

^a^*p *value less than 0.05 is significant; ^b^mean ± standard deviation; ^c^median (interquartile range). APACHE II, Acute Physiology and Chronic Health Evaluation II; SAPS II, Simplified Acute Physiology Score II.

**Table 2:** Referring specialty (developmental set)

Referring specialty	Survivors (*n *= 3,145)	Non-survivors (*n *= 1,433)
Internal medicine	343 (10.9%)	307 (21.4%)
Cardiology	192 (6.1%)	187 (13.0%)
Pulmonary diseases	91 (2.9%)	69 (4.8%)
Neurology	42(1.3%)	43 (3.0%)
Surgery	1,249 (39.7%)	627 (43.7%)
Cardiothoracic surgery	889 (28.3%)	97 (6.8%)
Neurosurgery	40 (1.3%)	16 (1.1%)
Other	299 (9.5%)	82 (5.7%)
Admission type		
Medical	737 (24%)	723 (51%)
Unplanned surgery	497 (16%)	323 (23%)
Planned surgery	1,861 (60%)	359 (26%)

### Classification tree

**Figure 1:**
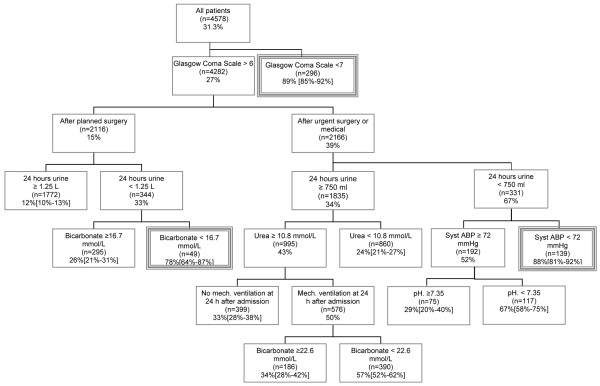
Classification tree to predict mortality before hospital discharge in patients 80 years old or older who were admitted to the intensive care unit. Percentages represent the likelihood of in-hospital mortality for patients in each subgroup (percentages in brackets represent 95% confidence interval). A subgroup with mortality risk of more than 75% is indicated by a double-framed box. Syst ABP, systolic ambulatory blood pressure.

The classification tree was obtained by binary recursive partitioning from the developmental data set and is presented in Figure 1. Note that a right branch always corresponds to the subgroup with the higher risk. Every patient fulfils the criteria of just 1 of the 11 mutually exclusive subgroups at the leaves of the trees. A leaf corresponds to a subgroup that is not further subdivided. The predicted likelihood to die before hospital discharge is given by the corresponding box. For example, all patients with a GCS score of more than 6, admitted to the ICU after planned surgery, and with urine production over the first 24 hours of more than 1.25 liters had a risk to die before hospital discharge of 11.8%. Likewise, all patients with a GCS score of less than 7 had a risk of 89.2% (Figure 1). Of all 4,578 patients in the developmental set, 435 (9.5%) had a risk higher than 85% and 484 patients (10.6%) had a risk higher than 70%.

### Performance of classification tree compared with original SAPS II and recalibrated SAPS II models

**Figure 2:**
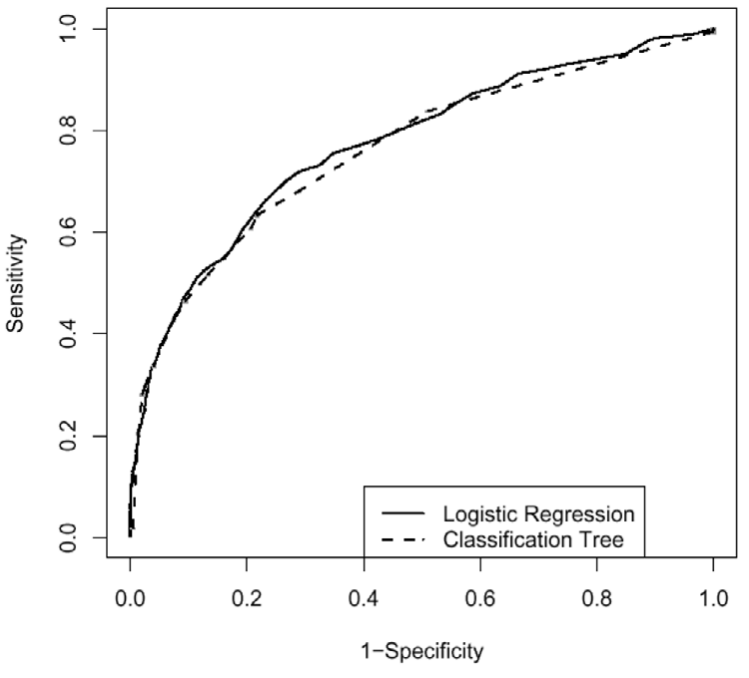
Receiver operating characteristic curves for the classification tree and the Simplified Acute Physiology Score II (SAPS II) model (logistic regression). The curves of the original SAPS II model and the recalibrated SAPS II model are identical.

Overall performance of the different models is shown in Tables 3 and 4. Discrimination (that is, the ability to distinguish between survivors and non-survivors) is given by the ROC-AUC (Figure 2). The accuracy of the predictions is given by the Brier score (that is, the mean squared difference between the prediction and the actual outcome of all patients); the lower the Brier scores, the higher the accuracy. When tested on all patients in the validation set, the ROC-AUC was 0.77 for all three models (Table 3). Also, identical Brier scores were found for the three models. However, the Brier score is sensitive to calibration and it showed that the recalibrated SAPS II model was better than the original SAPS II model (95% CI 0.0016 to 0.0081). The 95% CIs for the classification tree versus the original SAPS II model (-0.0172 to 0.011) and for the classification tree versus the recalibrated SAPS II model (-0.0119 to 0.0139) were not significantly different.

**Table 3:** Performance of classification tree, original SAPS II, and recalibrated SAPS II in all patients in the independent validation set (*n *= 2,289)

	Classification tree	SAPS II	Recalibrated SAPS II
ROC-AUC ± SD	0.77 ± 0.01	0.77 ± 0.01	0.77 ± 0.01
Brier score	0.16	0.16	0.16
Threshold PPV			
0.5	0.69 (0.64–0.73)	0.68 (0.64–0.72)	0.71 (0.67–0.76)
Died (*n*)	329	340	305
Predicted to die (*n*)	480	502	427
0.7	0.85 (0.8–0.89)	0.78 (0.73–0.82)	0.81 (0.76–0.86)
Died (*n*)	196	241	176
Predicted to die (*n*)	230	309	215
0.8	0.88 (0.83–0.91)	0.83 (0.77–0.87)	0.88 (0.81–0.92)
Died (*n*)	184	168	119
Predicted to die (*n*)	210	203	136

Confidence intervals (CIs) of differences between PPVs (asterisk indicates statistical significance at the 0.05 level) are as follows. For classification tree versus SAPS II, the CIs for cutoffs of 0.5, 0.7, and 0.8 are -0.032 to 0.047, 0.023 to 0.121*, and -0.006 to 0.104, respectively. For classification tree versus recalibrated SAPS II, the CIs for cutoffs of 0.5, 0.7, and 0.8 are -0.07 to 0.011, -0.027 to 0.086, and -0.057 to 0.055, respectively. For recalibrated SAPS II versus SAPS II, the CIs for cutoffs of 0.5, 0.7, and 0.8 are 0.056 to 0.016*, 0.072 to 0.004*, and 0.087 to 0.003*, respectively. PPV, positive predictive value; ROC-AUC, area under the receiver operating characteristic curve; SAPS II, Simplified Acute Physiology Score II; SD, standard deviation.

**Table 4:** Performance of classification tree, original SAPS II, and recalibrated SAPS II in patients in the independent validation set who fulfill the entry criteria of SAPS II model (*n *= 1,594)

	Classification tree	SAPS II	Recalibrated SAPS II
ROC-AUC ± SD	0.72 ± 0.01	0.75 ± 0.01	0.75 ± 0.01
Brier score	0.19	0.18	0.18
Threshold PPV			
0.5	0.64 (348)	0.66 (365)	0.69 (308)
0.7	0.83 (144)	0.76 (218)	0.79 (144)
0.8	0.85 (129)	0.80 (137)	0.84 (87)

PPV, positive predictive value; ROC-AUC, area under the receiver operating characteristic curve; SAPS II, Simplified Acute Physiology Score II; SD, standard deviation.

We also performed a Hosmer-Lemeshow test within 10 degrees of freedom. It resulted in an H statistic of 64.3 (*p *value < 0.00001) and a C statistic of 89 (*p *value < 0.00001) for the original SAPS II. For the recalibrated SAPS II, we found an H statistic of 9.5 (*p *value = 0.49) and a C statistic of 21.6 (*p *value = 0.02). The recalibrated SAPS II model is clearly much better.

To test the ability to identify high-risk patients, we calculated PPVs for three risk levels corresponding to the following cut-points: 0.5, 0.7, and 0.8 (Tables 3 and 4). When tested on all patients in the validation set (Table 3), the recalibrated SAPS II model had the highest PPV for the lowest risk with cut-point 0.5 (non-significant versus tree; significant versus original SAPS II). However, the classification tree had the highest PPV when patients were identified with a risk higher than 0.7 (PPV = 0.85, significant versus original SAPS II; non-significant versus recalibrated SAPS II) and higher than 0.8 (PPV = 0.88, non-significant differences with original and recalibrated SAPS II). The classification tree, the original SAPS II, and the recalibrated SAPS II model predicted a likelihood of more than 0.8 to die before hospital discharge in 210 (9.2%), 203 (8.9%), and 136 (5.9%) of 2,289 patients in the validation database, respectively.

### Performance of the models in patients fulfilling the entry criteria of the SAPS II model

The original SAPS II model excludes many patients for estimation of the risk to die. To make a fair comparison, we also tested the three models in the patients of the validation set fulfilling the criteria of the SAPS II model (Table 4). The most important group of patients that was excluded in this analysis corresponded to patients after cardiac surgery. Interestingly, overall performance, as measured by the ROC-AUC and the Brier score, was lower for all models compared with performance in the complete validation set. In this analysis, the number of patients with an estimated risk of higher than 0.8 and the PPV were largest for the classification tree model (no significant testing was attempted).

### Combination of classification tree and recalibrated SAPS II

We tested the hypothesis that combining the classification tree with the recalibrated SAPS II model would lead to a higher PPV for identifying high-risk patients. In the complete validation database, 112 (4.9%) of the patients had a predicted risk of mortality of more than 80% in both the classification tree and recalibrated SAPS II. Observed mortality in these patients was 105 (that is, with a PPV of 94%).

## Discussion

The results of this study show that it is possible to reliably identify a relatively high percentage of very elderly ICU patients who have a very high risk to die before hospital discharge. Up to almost 10% of patients were shown to have a risk to die of greater than 85%. Although overall predictive performance in all very elderly patients was similar for the SAPS II model, the PPV for high-risk subgroups was larger for the recalibrated SAPS II model and the classification tree, and the classification tree identified most patients at very high risk. This does not mean that classification tree-based models are better than logistic regression-based models. SAPS II was developed almost 20 years ago. We cannot rule out that a new model based on logistic regression and specifically developed for very elderly ICU patients would have even better predictive power. This classification tree offers the advantage that the predictions are based on only eight variables, making it very easy to use. Furthermore, it clearly shows which parameters are related to a bad outcome. The fact that low GCS scores appear to be most strongly related to death could prompt the finding of new treatment strategies for very elderly patients who are comatose. Another advantage of the classification tree is the symbolic representation, which is easier to interpret. RPA also automatically identifies the predictors, the cut-points, and the interactions among all possibilities. Furthermore, missing values are systematically dealt with.

To our knowledge, this is the first validated prognostic model based on recursive partitioning which is able to reliably identify high-risk mortality groups and which is developed and validated on a large group of patients. Our results are in line with other studies using a classification tree [15]. However, these studies were based on populations of patients described merely by malignancies or fitted on a small population without performing validation on a separate validation set [16-18].

Identification of high-risk groups of patients may be important for several reasons. First, as already mentioned, it focuses attention on groups of patients for whom current treatments may be insufficient. This in itself could lead to an improvement in care. Second, for some medical studies, enrollment of high-risk patients in clinical trials may provide the highest likelihood for finding a positive effect or facilitate investigating treatments with serious adverse side effects which are acceptable only if other treatments are not effective. Third, identification of high-risk subgroups may be used for case-mix correction when comparing the outcomes of very elderly patients in different ICUs. Fourth, it may be used for providing optimal information to patients, their relatives, and caregivers. Very elderly patients do not necessarily prefer intensive care treatment over palliative care that aims at comfort and pain relief. Interestingly, when presented hypothetical scenarios, patients state that they would decline intensive treatments if the likelihood of survival were very low [6].

There are some limitations to our findings. The classification tree-based model was developed in a Dutch population of very elderly ICU patients. Before this model can be used in other countries, it should be validated in an international population. Furthermore, the model is based on data from 1997 to 2003. Because the prognosis of ICU patients may change over time, repeated validation is necessary in the future if data from the model is to be used to support decision-making in individual cases. Also, the influence of providing prognostic information of this kind to individual patients is not known. In addition, do very elderly patients, when actually faced with a life-threatening condition, really prefer palliative care over life-sustaining treatments? Because they have decreased consciousness or are otherwise too ill, almost all very elderly patients with a very high risk to die are not able to express their preferences. Consequently, decisions regarding life-sustaining treatments are made by physicians and family members or other legal representatives [19,20]. Physicians are not always aware of the preferences of their seriously ill patients [21], and it is unknown to what extent end-of-life decisions by family members are influenced by the likelihood of survival [19,22]. For all these reasons, the use of prognostic models for decision-making in individual cases carries many dangers. They should not yet be used for this purpose, and more research is clearly necessary. Nevertheless, adequate communication, good decision-making, and respect for patients' autonomy are key determinants of patient and family satisfaction [23].

For economic reasons, prognostic models may also be used for triage decisions. Intensive care resources are limited and expensive [21]. It has been stated that, from an economic perspective, costs between $50,000 and $100,000 USD per year of life gained are acceptable in the US [24,25]. One could argue that ICU treatments should be given only to patients with a fair chance of survival [21]. However, because consensus is lacking about the likelihood of survival needed in order to offer ICU treatment to (very elderly) patients who otherwise would almost certainly die [26], we believe that current prognostic models should not be used for triage purposes.

In addition, other reliable parameters should be studied and added to current and soon-to-be-developed prognostic models. For instance, the presence of cognitive or functional impairment may play an important role in clinical decision-making in receiving life-sustaining treatment and therefore in prognosis [27]. But before adaptation of prognostic models is possible, more prospective studies need to be carried out to study the impact of pre-admission cognitive and functional impairment on short-term outcomes like ICU and hospital mortality or on long-term functional outcome, especially in the very elderly.

## Conclusion

Our results show that current prognostic models may reliably identify subgroups of very elderly patients who have a very high risk of dying before hospital discharge. We suggest that future research focus on how prognostic models may support individual patients and their families in decision-making to ensure that care is consistent with their preferences.

## Key messages

• Prognostic models reliably identify subgroups of very elderly ICU patients who have a high risk of dying before hospital discharge.

• Up to almost 10% of patients were shown to have a risk to die of greater than 80%.

• Overall predictive performance in all very elderly patients was similar for the SAPS II model, the recalibrated SAPS II model, and the classification tree model.

• In the very elderly, few predictors such as those used in the classification tree model resulted in performance similar to that of the SAPS II model.

• Identification of high-risk groups of patients may be important for several reasons.

## Abbreviations

APACHE = Acute Physiology and Chronic Health Evaluation; CI = confidence interval; GCS = Glasgow Coma Scale; ICU = intensive care unit; MPM II = Mortality Probability Models II; NICE = National Intensive Care Evaluation; PPV = positive predictive value; ROC = receiver operating characteristic; ROC-AUC = area under the receiver operating characteristic curve; RPA = recursive partitioning analysis; SAPS II = Simplified Acute Physiology Score II.

## Competing interests

The authors declare that they have no competing interests.

## Authors' contributions

SR was a principal investigator of the study, helped design the protocol and supervise its progress, and helped draft the manuscript. EJ was a principal investigator of the study, helped design the protocol and supervise its progress, was involved in the acquisition of the data, and helped draft the manuscript. AA-H was involved in the acquisition of the data and was responsible for the statistical analysis. ML helped draft the manuscript. All authors contributed to the interpretation of the data and revisions of the paper and read and approved the final manuscript.
